# Inequalities and risk factors related to non-participation in colorectal cancer screening programmes: a systematic review

**DOI:** 10.1093/eurpub/ckaa203

**Published:** 2020-12-12

**Authors:** Saloa Unanue-Arza, Maite Solís-Ibinagagoitia, Marta Díaz-Seoane, Isabel Mosquera-Metcalfe, Isabel Idigoras, Isabel Bilbao, Isabel Portillo

**Affiliations:** 1 Department of Nursing I, Faculty of Medicine and Nursing, University of the Basque Country UPV/EHU, Bizkaia, Spain; 2 BioCruces-Bizkaia Health Research Institute, Barakaldo, Spain; 3 Department of Preventive Medicine and Public Health, University Clinical Hospital of Valladolid, Valladolid, Spain; 4 Basque Country Colorectal Cancer Screening Programme, Osakidetza, Basque Health Service, Bilbao, Spain

## Abstract

**Background:**

Colorectal cancer (CRC) screening programmes require high levels of participation in order to reduce mortality. To improve participation rates, it is necessary to identify the health risk factors and social inequalities associated with non-participation.

**Methods:**

A systematic review was conducted between June and September of 2019 in six databases: CINHAL, Medline, Scopus, Social Sciences Citation Index, Embase and PsycINFO. Studies assessing the relationship between health risk factors, participation in preventive activities and participation in CRC screening were included. Methodological assessment was carried out according to the Quality Assessment Tools of the National Heart, Lung and Blood Institute.

**Results:**

A total of nine studies that analyze participation in both organized and opportunistic screening programmes using any type of screening method were finally selected. Data were mainly self-reported although in two studies medical records were also studied. We identified several variables: gender, body mass index, consultation with a doctor or a specialist, educational level, employment, health insurance, residence, ethnicity, age, marital status, income, other preventive activities, obesity, physical activity, smoking, family history of CRC and general health status.

**Conclusion:**

The scarcity of studies linking risk factors, social inequalities and participation in preventive activities for participation in screening in the same study makes it difficult to reach definitive patterns related to non-participation in CRC screening programmes. Nevertheless, being under 60, obese, smoker and sedentary have shown an association with non-participation as well as not visiting a doctor.

## Introduction

Behavioural risk factors, such as smoking, drinking too much alcohol, nutritional choices or physical inactivity, often acquired in childhood, can condition health status in adult life.^1^ Specifically, physical inactivity, a diet rich in red meat, low in fibre and low consumption of fruits and vegetables, smoking and a high body mass index have been shown to be associated with a worse health status of individuals.^2–4^ However, these factors can be modified throughout life, reducing the risk of several pathologies and improving overall health.^5^ People who have these behaviours often take less care of themselves, and therefore also participate less in screening programmes and other preventive activities.^6^ This is often because certain social determinants may condition their behaviour. In fact, socio-economic status and social inequalities are related to risk factors, indeed, the higher the socioeconomic level, the greater the presence of risk factors.^7^ Lower socio-economic status and educational level, worse employment conditions and place of residence are some of the social inequalities that can lead to not undergoing preventive activities such as regular health check-ups or participation in screening programmes.^7–10^

The implementation of colorectal cancer (CRC) screening programmes is widely recommended, in fact, both the United States Preventive Services Task Force and the Council of the European Union recognize its potential and recommend organized programmes.^11^^,^^12^ Furthermore, cancer screening programmes can reduce cancer-specific and all-cause mortality.^13^ Despite this, CRC screening programmes are not implemented equally around the world, depending on the CRC incidence, economic resources and healthcare structure.^14^ Usually, they differ in the screening method—Faecal Occult Blood Test (FOBT), optical sigmoidoscopy, optical colonoscopy or computed tomography colonoscopy—the organizational characteristics—population-based or opportunistic—and the target population. Moreover, high participation rates are essential in screening programmes in order to be cost effective and to achieve health benefits.^15–17^ Therefore, knowing how both social inequalities and participation in preventive activities influence participation CRC screening specifically could help improving participation rates.

The goal of this study is to identify the association between non-participation in CRC screening programmes and social inequalities and risk factors or participation in preventive activities.

## Methods

### Data sources

A systematic review of the literature was conducted between June and September of 2019 using the following databases: CINHAL, Medline, Scopus, Social Sciences Citation Index, Embase and PsycINFO.

### Search strategy

The search strategy combined a wide range of Medical Subject Headings (MeSH) and free text terms related to screening, CRC, participation and social inequalities. This search was limited to articles published from 2000 to June 2019. The search strategy used in Medline is given in Supplementary table S1.

### Screening and review process

Studies were included according to this criteria: (i) studies focusing on risk factors, participation in preventive activities (preventive health check-ups or participation in screening programmes), inequalities according to the Dahlgreen and Whithead model^18^ and participation in any type of CRC screening programmes with any type of screening method (public or private health system); (ii) primary studies; (iii) studies in which 45–75-year-old people at average -risk of CRC were offered to screening; (iv) published in peer-reviewed journals between 2000 and 2019 in English, French, Portuguese and Spanish; (v) where relationships between social inequalities and risk factors or participation in preventive activities and participation in CRC screening were analyzed and whose quality was fair or good.

Two authors screened all titles and abstracts of the identified references, with a third in case of discrepancy. Then, the whole text of the selected studies was analyzed for eligibility according to inclusion and exclusion criteria. Disagreements were resolved by consensus. Reviewers eliminated articles not related to the study as shown in Figure 1. This systematic review was performed according to the Preferred Reporting Items for Systematic Reviews and MetaAnalyses (PRISMA) guidelines.^19^

**Figure .**
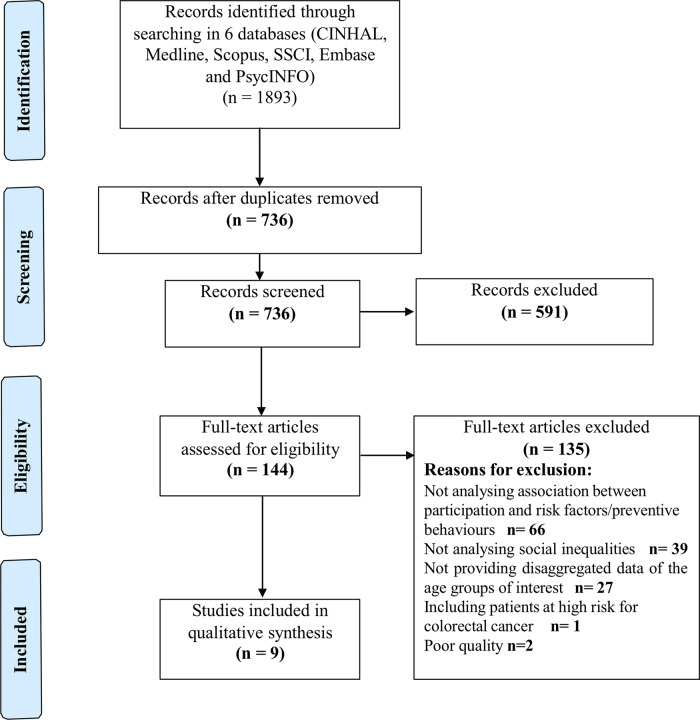
PRISMA flowchart

Two independent authors extracted data, including the name of the first author, year of publication, country and region, methodology, exposure and main results. The description of the included studies is shown in table 1. Variables are presented as percentages for participation as odds ratios (ORs) and 95% confidence intervals.

**Table 1:** Description of the included studies

Author, year, country/region	Methodology	Exposure (if applicable, number of categories)	Main findings^a^	Quality of the study^b^
Anderson 2011^27^ Connecticut (USA)	Study design: observational retrospective cross-sectional study Participants *N* = 354 underinsured patients Age: 50–74 years Gender: 62% female Type of screening: opportunistic Screening method: free colonoscopy Data source: medical record and self-reported questionnaire Statistical analysis: multivariate analysis (*P* < 0.05) Subgroup analysis: obese and non-obese	CRC risk factor/preventive activity: Age (continuous variable), obesity (2), smoking status (3), diabetes (2), aspirin (2), family history of CRC (2), family history of CRC (2), past CRC screening test (2) Inequality indicator: Education (2), gender (2), ethnicity (3), English language (2), employment (2), next of kin (4)	Screening participation rate (medical record): 74.3% Main outcome: non-participation Obesity [OR = 2.16 (1.20–3.89)] was associated with non-participation Obese men [OR = 2.27 (1.04–4.55)] were as likely to be non-participant as obese women [OR = 2.70 (1.2–4.54)] High school graduate [OR = 0.55 (0.30–0.98)] Ref: no Next of kin: spouse/significant other [OR = 0.18 (0.07–0.48)]; friend [OR = 0.23 (0.08–0.70)]; other family [OR = 0.22 (0.08–0.59)] Ref: none	Fair
Bertaut 2018^21^ Côte-d’Or (France)	Study design: observational retrospective cross-sectional study Participants *N* = 1856 (female that participated in breast cancer screening) Age: 50–65 years Gender: only female Type of screening: organized Screening method: FOBT Data source: self-reported questionnaire Statistical analysis: multivariate polytomic regression (*P* < 0.15)	CRC risk factor/preventive activity: Age (2), cervical cancer screening participation (2), BMI (3), family history of colorectal cancer (3), influenza vaccine (2), GP consultation in the past 12 months (2), gynaecologist consultation in the past 12 months(2), gastroenterologist consultation in the past 12 months (2), physical activity practice (2), tobacco smoking (2), alcohol (2), fruit and vegetable consumption (3) Inequality indicator: social and occupational group (4), marital status (2), Diploma (5), Supplementary health insurance (2)	Screening participation-rate (FOBT in the previous two years): 56.6% Main outcome: participation in breast, cervical and colorectal cancer screening. Participation in breast and colorectal cancer screening vs. participation in all screenings: BMI≥30 OR = 2.22 (1.34–3.70) Ref: <25 Gynaecologist consultation in the past 12 months OR = 0.09 (0.05–0.14) Ref: no	Good
Dimitrakaki 2009^22^ Greece	Study design: observational retrospective cross-sectional study Participants *N*: 297 Age: 50–69 years Gender: 57% female Type of screening: opportunistic Screening method: FOBT Data source: Hellas Health I National Survey (self-reported questionnaire) Statistical analysis: multivariate logistic regression (*P* < 0.05)	CRC risk factor/preventive activity: Age (3), smoking (2), general health (3), participation in other screening programmes (4) Inequality indicator: residence (2), family doctor (2), education level (3), social class (3), insurance (2)	Screening participation rate: 9.3% in women, 10.9% in men. Main outcome: participation in FOBT in the last three years Female Have a family doctor OR = 5.55 (1.30–23.68) Ref: no Male Non-significant associations	Fair
Fon Sing 2013^28^ France	Study design: observational retrospective cross-sectional study Participants *N*: 2276 Age: 50–74 years Gender: 52.5% female Type of screening: organized Screening method: guaiac FOBT Data source: French Health-care and Insurance Survey Statistical analysis: multivariate logistic regression (*P* < 0.1)	CRC risk factor/preventive activity: Age (5), having consulted a medical specialist in the last 12 months (3), alcohol consumption (4), having had a mammogram (3), having had a Pap smear (3), Self-reported dental status (4), self-reported health status (4), tobacco consumption (5) Inequality indicator: gender (2), current or last occupation (5), healthcare renouncement (4), district with colorectal cancer screening programme (3), highest educational level reached (5), living in rented accommodation (3), monthly household income per consumer unit (4), healthcare access benefiting a free additional health insurance for people with low income (3), having a private additional health insurance (3) 100% coverage for medical fees for a long-term disease (2)	Screening participation rate in the last two years: 42% No differences according to gender Main outcome: participation in g-FOBT in the last two years (self-reported) Female: Age 55–59 OR = 2.0 (1.35–2.97); 60–64 OR = 2.71 (1.80–4.09); 65–69 OR = 5.58 (3.40–9.14); 70–74 OR = 3.98 (2.36–6.70) Ref: 50–54 Pilot district OR = 1.84 (1.74–2.46) Ref: other districts Additional health insurance OR = 2.01 (1.09–3.73) Ref: no Having had a mammogram OR = 3.31 (2.31–4.75) Ref: never or more than two years ago Having had a Pap smear OR = 1.59 (1.16–2.19) Ref: never or more than three years ago Male: Age 60–64 OR = 2.55 (1.66–3.91); 65–69 OR = 3.84 (2.30–6.41); 70–74 OR = 2.46 (1.45–4.17) Ref: 50–54 100% coverage for medical fees for a long-term disease OR = 1.61 (1.14–2.27) Ref: yes Pilot district OR = 1.67 (1.25–2.24) Ref: other districts Additional health insurance OR = 6.53 (3.52–12.12) Ref: no Having consulted a medical specialist in the last 12 months OR = 1.67 (1.22–2.29) Ref: no Tobacco consumption: Former smoker OR = 2.58 (1.57–4.23); never smoked OR = 2.98 (1.81–4.90) Ref: current smoker with a high tobacco consumption	Good
Katz 2015^25^ Ohio (USA)	Study design: observational retrospective cross-sectional study Participants *N*: 637 Age: 51–75 years Gender: only female Type of screening: opportunistic Screening method: FOBT, colonoscopy and sigmoidoscopy Data source: self-reported questionnaire Statistical analysis: multivariable logistic regression (*P* ≤ 0.1)	CRC risk factor/preventive activity: Age, self-rated health (2), smoking status (2), medical condition requiring medical doctor visits (2), checkup in last two years (2), doctor recommendation for all three cancer screening tests (2) Inequality indicator: ethnicity (2), marital status (2), education(2), annual household income(3), employment status (3), private insurance (2)	Self-referred screening participation rate: 30.1% (across imputed datasets). Main outcome: participation in breast, cervical and colorectal cancer screening. Annual household income: >60 000 $/year [OR = 3.53 (1.49–8.33)] Ref: <30.000 $/year Medical condition requiring medical doctor visits OR = 3.16 (1.29–7.74) Ref: No Retired/volunteer: OR = 3.16 (1.07–9.33) Ref: unemployed/disabled	Fair
Knudsen 2017^23^ South East Norway	Study design: observational retrospective cross-sectional study Participants *N*: 3114 Age: 50–74 years Gender: 53% female Type of screening: organized Screening method: FOBT Data source: self-reported questionnaire Statistical analysis: multivariable logistic regression. (*P* ≤ 0.1)	CRC risk factor/preventive activity: Age (5), smoking (4), BMI (5), physical activity (5), alcohol (6), diet score (5) Inequality indicator: gender (2), centre (2), occupation (5), education length (4), marital status (3), ethnic background (3)	Screening participation rate: 83% Main outcome: participation in first and second round Current smoking OR = 2.01 (1.24–2.1) Ref. non-smoking BMI > 35 OR = 2.01 (1.25–3.24) Ref: BMI 16.9–24.9 Physical activity (third quartile) OR = 0.70 (0.52–0.94) Ref: first quartile Female: Age 50–54 OR = 2.09 (1.01–4.32); 55–59 OR = 2.20 (1.08–4.50). Ref: 70–74 Current smoker OR = 1.67 (1.15–2.43) Ref: never smoked BMI 30–35 [OR = 1.54 (1.00–2.37)]; >35 [OR = 1.94 (1.03–3.65)]. Ref: 16.9–24.9 Thirty minutes of physical activity: 4–6.5 times/week [OR = 0.65 (0.42–0.98)] Ref: twice/week Male: Occupation: disable/on rehabilitation [OR = 1.65 (1.05–2.57)] Ref: working Non-native ethnic background (OR = 1.66 (1.00–2.79) Ref: native background Current smoker [OR = 1.60 (1.09–2.35)] Ref: never smoked BMI > 35 [OR = 2.09 (1.00–4.40)] Ref : 16.9–24.9 kg/m^2^	Good
Seibert 2017^26^ USA	Study design: observational retrospective cross-sectional study Participants *N*: 8550 Age: 50–75 years Gender: 55% female Type of screening: opportunistic Screening method: FOBT, colonoscopy and sigmoidoscopy Data source: National Health Interview Survey Statistical analysis: multivariable logistic regression (*P* ≤ 0.05)	CRC risk factor/preventive activity: Obesity (5), guideline adherence (2), screening method (2) Inequality indicator: Gender (2)	Screening participation rate: 58.9% Main outcome: participation in any CRC screening method Female: No association Male: Guideline adherence: obese grade III (OR = 0.35 (0.17–0.75), *P* < 0.001) Ref: normal weight Endoscopic screening method: obese grade III [OR = 0.37 (0.18–0.69)] Ref: normal weight	Good
Senore 2010^20^ Italy	Study design: Population-based controlled trial Randomized: yes Masking: no Multicentre: yes (five centres) Participants *N*: 26 255 Age: 55–64 years Gender: 53% female Recruitment period: non-available Type of screening: organized Screening method: FOBT and sigmoidoscopy Data source: self-reported survey Statistical analysis: multivariable logistic regression (*P* < 0.05)	CRC risk factor/preventive activity: Age (2), knowledge of personal risk (3), family history of CRC (3), physical activity (2), smoking habits (3), screening attitude (3), GP's advice (2), health status (2), reading information material (3), knowledge of CRC preventive test (2) Inequality indicator: Gender (2), education (3), employment status (2), source of information (2)	Screening participation rate: 47.2% Main outcome: participation in any CRC screening method. Both genders Family history of CRC [OR = 3.62 (2.02–6.49)] Ref: no history of CRC Physical activity (≥1/month) [OR = 1.85 (1.33–2.55)] Ref: no physical activity Smoking habits: current smoker [OR = 0.68 (0.47–0.98)] Ref: never smoked Screening attitude: believes screening is effective-anxiety [OR = 0.32 (0.23–0.45)]; believes screening is ineffective [OR = 0.12 (0.08–0.19)]. Ref: believe screening is effective-no anxiety GP’s advice [OR = 4.24 (3.11–5.78)]. Ref: did not seek GP counselling Health status: fair/poor [OR = 0.71 (0.52–0.96)]. Ref: good health status Employment status: employed [OR = 0.78 (0.66–0.93)]. Ref: housewife/retired Reading information material: read the letter and the leaflet [OR = 3.18 (2.12–4.76)]; read the letter [OR = 1.85 (1.23–2.78)]. Ref: did not read the letter or the leaflet Does know the test [OR = 0.49 (0.34–0.70)]. Ref: does not know the test Female First-degree relative with CRC [OR = 4.61 (2.09–10.13)] Ref: no Regular screening using mammography [OR = 3.73 (1.15–12.12)] Ref: No Male Physical activity: ≥1/month [OR = 2.33 (1.32–4.13)]. Ref: no physical activity	Fair
Sicsic 2014^24^ France	Study design: observational retrospective cross-sectional study Participants *N*: 12 156 Age: 50–74 years Gender: 51% female Type of screening: organized Screening method: FOBT Data source: French Health Care and Health Insurance Survey Statistical analysis: multivariable logistic regression (*P* < 0.05)	CRC risk factor/preventive activity: Age (4), chronic disease (2), self-rated health (4), number of consultations with a GP (2), number of consultation with a specialist (2), tobacco consumption (4), alcohol (4) Inequality indicator: gender (2), social class (8), marital status (2), complementary health insurance (3)	Screening participation rate (2010): 38.9% Main outcome: non-participation in the CRC screening programme Female: Number of consultations with a GP: two or less OR = 1.02 (1.18–1.36) (Ref: three or more) No consultation with a specialist [OR = 1.68 (1.45–1.96)] Ref: one or more Tobacco: heavy smoker [OR = 1.68 (1.23–2.28)] Ref: non smoker Alcohol: non-drinker [OR = 1.22 (1.05–1.42)] Ref: safe consumer Male: Social class: farmer [OR = 1.46 (1.08–1.99)]; non-skilled worker [OR = 1.60 (1.23–2.08)] Ref: associated profession No complementary health insurance [OR = 1.40 (1.00––1.97)] Ref: private insurance Number of consultations with a GP: two or less [OR = 1.22 (1.06–1.41)] Ref: three or more No consultation with a specialist [OR = 1.29 (1.12–1.49)] Ref: one or more Tobacco: heavy smoker [OR = 1.70 (1.31–2.22)], light smoker [OR = 1.28 (1.03–1.59)] Ref: non- smoker	Good

CRC, colorectal cancer; OR, odds ratio; BMI, body mass index; GP, general practitioner; Ref, reference; FOBT, Faecal Occult Blood test; g-FOBT, Guaiac Faecal Occult Blood test; Ref., reference category.

a Only statistically significant results after adjustment by the other significant variables were described.

b Quality assessment according to the National Heart, Lung, and Blood Institute Study Quality Assessment Tools.

### Study methodological quality assessment

Methodological assessment was carried out independently in parallel by three researchers according to the Quality Assessment Tools of the National Heart, Lung and Blood Institute for quantitative studies to judge each study in terms of “good,” “fair” or “poor” quality.^20^ If the ratings differed, then reviewers discussed the article and a final decision was made in an attempt to reach consensus.

## Results

### Literature search

In total, 1893 studies were identified in the bibliographic search and after removing duplicates, 736 were screened for title and abstract. From those, 144 full-text articles were assessed for eligibility and 135 were excluded according to the following reasons: the study did not analyze the relationship between preventive activities or risk factors and social inequalities individually with participation in CRC screening programmes, did not study 45–75-year-old population, studied high-risk population or the methodological quality was poor. After evaluating the full text, nine articles were included in the systematic review. The list of excluded articles is shown in Supplementary table S2.

### Quality and characteristics of the selected studies

All studies included in this review were descriptive and observational retrospective cross-sectional except one,^21^ which was a randomized multicentre population-based controlled trial. Five of the studies were conducted in organized screening programmes,^21–25^ whereas the rest were opportunistic,^26–29^ using different tests. In four of them, only FOBT was used,^22^^,^^24^^,^^25^^,^^27^ while in three of them,^21^^,^^28^^,^^29^ colonoscopy and/or sigmoidoscopy were offered to the people invited, in addition to FOBT as a screening test. According to the age of the invited people, four studies included 50–74/75-year-old population^23–26^^,^^29^ and the rest studied people from 50 to 65^22^ or 69^27^ whereas in only one study the population was 55–64.^21^ Finally, in one study the test used was the guaiac FOBT (FOBTg),^23^ and in another one the colonoscopy exclusively.^26^ In all the studies, the analyzed data were self-reported and, in addition, in three of them,^23^^,^^25^^,^^29^ national survey data were added and in two studies medical records.^26^^,^^27^ Regarding the gender of the persons included in the studies, it should be noted that only women were included in two of them.^22^^,^^28^ Three of the studies were conducted in France,^22^^,^^23^^,^^25^ three in the United States,^26^^,^^28^^,^^29^ and the other three in Greece, ^27^ Italy^21^ and Norway.^24^

Of the nine studies finally included, according to the aforementioned Quality Assessment Tools for quantitative studies,^20^ five were of good quality (the least risk of bias whose results are considered to be valid)^22–25^^,^^29^ and four of fair quality (susceptible to some bias deemed not sufficient to invalidate its results).^21^^,^^26–28^ The results of the quality assessment are shown in Supplementary tables S3 and S4. The strengths of the observational included are the clarity of the objective and description and selection of the study population, the different levels of exposure and their measurement regarding the factors studied and the measurement of the results. On the other hand, the main weaknesses were that the sample size was not adequately justified. Due to the characteristics of the studies, criteria 10 and 13 were not applicable for descriptive studies. The major limitation of the intervention study was the absence of blinding in the measurement of the outcome variable. We eliminated two studies due to their low methodological quality, however, both include variables that have also been collected in the studies included in this review.

### Synthesis of evidence

The main results of the included studies are described below according to the factors that may be related to participation in CRC screening programmes grouped into social inequalities, participation in preventive health activities and risk factors.

It is necessary to emphasize that there is a high variability in the studied variables among the selected studies. The factors analyzed in each study are shown in Table 2.

**Table 2:** Factors analyzed in each of the included studies

	Studies
Health risk factors	
Age	Fon Sing et. al 2013, Katz et al. 2015, Knudsen et al. 2017, Senore et al. 2010, Sicsic et al. 2014
Alcohol	Bertaut et al. 2018, Fon Sing et al. 2013, Knudsen et al. 2017, Sicsic et al. 2014
Aspirin	Anderson et al. 2011
Chronic disease	Sicsic et al. 2014
Dental status	Fon Sing et al. 2013
Diabetes	Anderson et al. 2011
Diet	Knudsen et al. 2017
Family history of CRC	Anderson et al. 2011, Bertaut et al. 2018, Senore et al. 2010
First degree relative CRC	Sicsic et al. 2014
Fruit and vegetable consumption	Bertaut et al. 2018
General health status	Dimitrakaki et al. 2009, Fon Sing et al. 2013, Katz et al. 2015, Katz et al. 2015, Senore et al. 2010, Sicsic et al. 2014
Knowledge of personal risk	Senore et al. 2010
Obesity/BMI	Anderson et al. 2011, Bertaut et al. 2018, Knudsen et al. 2017, Seibert et al. 2017
Physical activity	Bertaut et al. 2018, Knudsen et al. 2017, Senore et al. 2010
Smoking status	Anderson et al. 2011, Bertaut et al. 2018, Dimitrakaki et al. 2009, Fon Sing et. al 2013, Katz et al. 2015, Knudsen et al. 2017, Senore et al. 2010, Sicsic et al. 2014
Participation in preventive activities	
Cervical cancer screening participation	Bertaut et al. 2018
Consultation with a GP	Bertaut et al. 2018, Sicsic et al. 2014
Consultation with a specialist	Bertaut et al. 2018, Fon Sing et al. 2013, Sicsic et al. 2014
Doctor recommendation	Katz et al. 2015, Senore et al. 2010,
Guidelines adherence	Fon Sing et. al 2013, Seibert et al. 2017, Senore et al. 2010, Sicsic et al. 2014
Influenza vaccine	Bertaut et al. 2018
Participation in other screening programmes	Dimitrakaki et al. 2009, Fon Sing et al. 2013
Past CRC screening test	Anderson et al. 2011
Social inequalities	
Additional health insurance	Fon Sing et. al 2013, Sicsic et al. 2014
Education	Anderson et al. 2011, Bertaut et al. 2018, Dimitrakaki et al. 2009, Fon Sing et. al 2013, Katz et al. 2015, Knudsen et al. 2017, Senore et al. 2010
English Language	Anderson et al. 2011
Gender	Anderson et al. 2011, Dimitrakaki et al. 2009, Fon Sing et al. 2013, Knudsen et al. 2017, Seibert et al. 2017, Senore et al. 2010, Sicsic et al. 2014
Having a family doctor	Dimitrakaki et al. 2009, Fon Sing et. al 2013, Sicsic et al. 2014
Health insurance	Bertaut et al. 2018, Dimitrakaki et al. 2009, Fon Sing et. al 2013, Katz et al. 2015, Sicsic et al. 2014
Income	Fon Sing et. al 2013, Katz et al. 2015
Knowledge of the CRC preventive test	Senore et al. 2010
Living in a rented accommodation	Fon Sing et al. 2013
Marital status/next of kin	Anderson et al. 2011, Bertaut et al. 2018, Katz et al. 2015, Knudsen et al. 2017, Sicsic et al. 2014
Occupation	Anderson et al. 2011, Bertaut et al. 2018, Fon Sing et. al 2013, Katz et al. 2015, Knudsen et al. 2017, Senore et al. 2010
Ethnicity	Anderson et al. 2011, Katz et al. 2015, Knudsen et al. 2017
Reading information material	Senore et al. 2010
Place of residence	Dimitrakaki et al. 2009, Fon Sing et. al 2013, Knudsen et al. 2017
Social class	Bertaut et al. 2018, Dimitrakaki et al. 2009, Sicsic et al. 2014
Source of information (reading newspaper)	Senore et al. 2010

CRC, colorectal cancer; GP, general practitioner.

## Social inequalities

Gender was analyzed in seven of the nine studies^21^^,^^23–27^^,^^29^ (in three of them, the analysis was stratified^25^^,^^27^^,^^29^) and in the other two, only women were included.^22^^,^^28^ In studies that analyzed both genders, the percentage of women was between 51% and 62%. Five studies analyzed whether gender was related to participation in screening,^21^^,^^23^^,^^24^^,^^26^^,^^29^ and it was only in the Norwegian study that this relationship was established,^24^ concluding that women participated more than men.

In addition, five studies analyzed all possible factors related to participation by gender.^23–25^^,^^27^^,^^29^ For women, having a family doctor,^27^ district,^23^ additional health insurance,^23^ age,^23^^,^^24^ smoking status,^24^^,^^25^ BMI,^24^ physical activity,^24^ consultations with a General Practitioner (GP),^25^ no consultation with a specialist,^25^ alcohol,^25^ first degree relative with CRC,^25^ regular screening using mammography^23^^,^^25^ and having a Pap smear^23^ were the analyzed variables. As far as men are concerned, obesity,^26^ age,^23^ health insurance,^23^ district,^23^ additional health insurance,^23^^,^^25^ having consulted a medical specialist,^23^^,^^25^ smoking status,^23–25^ occupation,^24^ non-native ethnic background,^24^ BMI,^24^ guideline adherence,^29^ endoscopic screening method,^29^ social class,^25^ number of consultations with a GP^25^ and physical activity were analyzed.^21^

Only in two studies was the relationship between all these factors established without any stratification by gender. Thus, whereas Anderson et al.^26^ reported an association between obesity, having a higher level of education and living with a partner with participation, Senore et al.^21^ did so with having a family history of CRC, physical activity and smoking.

When only women were included in the studies, participation was associated with BMI,^22^ having consulted with a gynaecologist in the past 12 months,^22^ annual income, state of health involving medical visits and being retired.^28^

Ethnicity has been analyzed in three studies,^24^^,^^26^^,^^28^ and only Knudsen et al.^24^ established that having a non-native ethnic background was associated with a higher probability of participation than natives for men in their study in Norway with FOBT, the association was not significant in women.

Anderson et al.^26^ reported the association between non-participation in the colonoscopy-based opportunistic screening programme and educational level, thus having high school diploma is associated with greater participation in both genders. Neither Fon Sing et al.^23^ found an association, although in this case the screening test was FOBTg. It should be noted that no association was found in any of the studies in which the screening test was FOBT.^21^^,^^22^^,^^24^^,^^27^^,^^28^

Regarding the employment situation, Katz et al.^28^ noted that being retired or being a volunteer is associated with participation in three recommended cancer screening programmes (breast, cervical and colorectal) with reference to the unemployed or disabled. On the other hand, Senore et al.^21^ established that, for both genders, employed people participated in a programme based on FOBT and sigmoidoscopy more than those who were unemployed.

Having health insurance has also been analyzed^22^^,^^23^^,^^25^^,^^27^^,^^28^ and an association between having additional health insurance and a higher probability of participating in CRC screening was only found in the studies by Fon Sing et al. and Sicsic et al.^23^^,^^25^ using FOBTg and FOBT, respectively.

The place where people live has also been considered,^23^^,^^24^^,^^27^ but only Fon Sing et al.^23^ found an association: those living in a district where the pilot programme was implemented have a higher probability of participating.

Although the relationship between marital status and participation was considered in four studies, it was not established in any of them.^21^^,^^23–25^

Income was analyzed in two studies,^23^^,^^28^ and a relationship was only established with participation in Katz et al.^28^ Those who had the highest household income (>60 000 dollars/year) were more likely to participate than those who had the lowest income (<3000 dollars/year) in an opportunistic screening programme with FOBT, colonoscopy or sigmoidoscopy.

## Participation in other preventive activities

Uptake in other preventive activities or use of the health system’s resources, mainly those related to the various screening programmes^22^^,^^23^^,^^26^^,^^27^ or visits to the GP^22^^,^^25^^,^^28^ or specialist^22^ have been studied. However, although Anderson et al.^26^ studied the relation between having taken a past CRC screening test, they found no association with participation in colonoscopy-based CRC screening.

Dimitrakaki et al.^27^ took into account participation in prostate and breast cancer screening programmes and did not observe any association with participation in CRC screening. However, Fon Sing et al.^23^ concluded that having a mammogram or Pap smear test was associated with a higher probability of participating in CRC screening (FOBT) than those who had never taken it, or had not taken it in the last 2 or 3 years, respectively.

On the other hand, concerning visits to the GP,^22^^,^^25^^,^^28^ gynaecologist^22^ or specialist,^23^^,^^25^ different results were reported. Bertaut et al.^22^ observed a relationship between participation and visiting the gynaecologist but not with visits to the GP or gastroenterologist. On the contrary, visiting the GP^25^^,^^28^ or specialist^23^^,^^25^ increased the probability of participating, while it was only established among men in the Fon Sing et al.^23^ study.

## Risk factors

The relationship between obesity and participation had been analyzed in four studies.^22^^,^^24^^,^^26^^,^^29^ Seibert et al.^29^ and Knudsen et al.^24^ discontinuation was analysed in two of them.^24^^,^^25^ Two studies analyzed discontinuation and in both they found an association with BMI. Knudsen et al.^24^ found that both men and women with a BMI over 35 are more likely to discontinue than people with normal weight (OR = 2.09 and OR = 1.94, respectively)^24^ while Seibert et al.^29^ only found an association in men with a BMI over 40 (adherence OR = 0.35) Only Anderson et al.^26^ analyzed the influence of obesity on participation and noted that people with a BMI greater than 30 are at greater risk of not participating in screening.

The results of the influence of physical activity are diverse, while Bertaut et al.^22^ found no association Knudsen et al.^24^ established a relationship with discontinuing CRC screening (OR = 0.7) although it was only stated in women in the stratified analysis. In turn, Senore et al.^21^ concluded that physical activity is only related to participation in men. In both studies, the higher the level of physical activity, the greater the likelihood of participating.

Tobacco has been widely studied,^21–28^ although only four studies established an association with participation.^21^^,^^23–25^ Two studies concluded that being a smoker decreased the probability of participating,^21^^,^^24^ while Sicsic et al.^25^ only established that relationship in women and Fon Sing et al.^23^ in men. On the contrary, only four studies analyzed the relationship between participation and alcohol consumption^22–25^ although it was only established in one of them, in fact, safe consumption^30^ was associated with a greater probability of participating.^25^

Moreover, although having a family history of CRC had been analyzed in three studies,^21^^,^^22^^,^^26^ but only among women, a greater probability of participating was established.^21^

Regarding general health status, although it had been analyzed in four studies, only Senore et al.^21^ established an association. In fact, worse health status was associated with a higher probability of non-participation.

Finally, it must be noted that diabetes and acetylsalicylic acid consumption,^26^ eating fruits and vegetables regularly^22^^,^^24^ and dental status^23^ had also been studied but none of the studies established a statistically significant relationship between these factors and participation.

## Discussion

The literature related to health risk factors and non-participation in CRC screening programmes is scarce. Moreover, it is even scarcer when social inequalities are also considered.

It has been observed that certain characteristics can increase the risk of not participating in screening even though there is not a clear pattern. Nevertheless, some risk factors have been identified in this review such as being under 60 years old, obese, smoker and sedentary as risk factors for not participating in the CRC screening programme. As far as age is concerned, it has emerged as a risk factor for not participating in the studies in which the analysis is not stratified; indeed, in some of the studies in which the analysis is stratified by gender,^22^^,^^23^ the risk is doubled and even quintupled with ORs ranging from 2.0 to 5.58.^23^ Obesity is also a factor to be considered, especially the BMI over 35, as it affects not only participation but also adherence. Smoking has also been shown to be a risk factor for not participating in screening, namely, Senore et al.^21^ in their clinical trial concluded that smoking prevented people from participating (OR = 0.68). Finally, physical activity has also been shown to be a factor, mainly with regard to adherence to screening.^21^^,^^24^

In addition, it is necessary to highlight how gender influences the other factors. It should be noted that some of the risk factors or preventive activities influence men and women differently, as can be seen from the stratified analysis by gender.^20^^,^^22–24^^,^^26^^,^^28^ In fact, Senore et al.,^20^ in the only clinical trial included in this review, conclude that despite several factors affecting participation these are considerably reduced in the stratified analysis. This should be taken into consideration when developing specific strategies to promote participation by decreasing the influence that gender may have. In accordance with our results, several studies have stated that people who attend primary healthcare infrequently, generally have poor concern about their health and do not participate in preventive activities.^17^^,^^31^^,^^32^ Visiting the GP or specialist increases the likelihood of participation, indeed, Zapka et al.^17^ stated that people who consider their health as good/excellent are less likely to participate than those who consider it acceptable/poor.

What is more, our results are consistent with other studies conducted from a qualitative approach in which the socio-cultural context of the place where the invited person lives (having a poor social support, not having a partner, having a low level of education or having a low income…) increases the risk of non-participation.^33^^,^^34^ The type of work (manual or non-manual), precariousness, not being able to go to the doctor during working hours, etc. may pose barriers to participation.^35^

The main limitation of this review is the heterogeneity of studies, regarding the lack of interventional studies, substantial variability for the studied factors, characteristics of the screening programmes, location, health system, employment, etc. Concerning the countries where the studies have been carried out, it should be noted that the characteristics of the health system and the screening programme itself and the socio-economic level of the people involved might influence the variability of the results. In fact, participation in Norway is much higher than in France, where screening is organized. A similar situation exists in the USA and Greece, where participation is lower maybe due to opportunistic screening. With these disparities, in addition to the factors mentioned, there could be other factors, cultural or related to trust in the health system, which also influence participation and have not been taken into account in these studies. Nevertheless, this heterogeneity allows us to design studies in which the same or similar variables can be studied, in order to compare them. On the other hand, it should be borne in mind that the data had been collected from self-administered questionnaires in several studies. With regard to the quality of the included studies, they do not indicate whether the factors that may influence participation were prior to screening which could help to establish a cause–effect relationship as well as an association. A more detailed description of the rationale for sample size and effect size is also missing. Studies that improve these two methodological aspects would be very useful for designing policies or interventions to increase participation in this and other screenings. In turn, the main strength of this review is that it provides a wide overview of the factors that may be involved in non-participation, including individual characteristics and habits related to health and social factors.

Considering that characteristics of the health system, universal coverage and an organized screening programme, facilitate participation.^36^^,^^37^ and that having unhealthy behaviours increases the risk of non-participation, basing screening on Primary Healthcare may be the key to improving participation as well as modifying unhealthy lifestyle habits. In fact, it is in Primary Healthcare where it is possible to address the determinants of health by implementing specific policies and actions and to empower people to improve their health by tackling risk factors, behaviours and inequalities that we have seen influence participation in CRC screening.^38^ Primary Healthcare could play a key role in encouraging patients to adopt healthier behaviours by identifying risk factors and promoting access to the health system. Nevertheless, more interventional studies are needed in this area to analyze in depth how all these factors influence participation and each other and to be able to design interventions aimed at inequalities or risk factors with specific approaches.

## Conclusions

In summary, the relationship of health risk factors with non-participation in CRC screening programmes taking into account social inequalities has not been extensively analyzed. Consequently, the results observed so far do not allow a pattern to be established. The heterogeneity on the design of the screening programmes in different countries as well as the differences in their health systems make it difficult to compare the results. Being under 60 years old, obese, smoker and sedentary have been considered as risk factors for not participating in the colon cancer screening programme. Gender had not shown statistically significant differences. Regardless of the type of health system, visiting a doctor implies an awareness of self-care and is therefore related to a lower risk of non-participation in cancer screening programmes. Inequalities affect lifestyles and risk factors, despite this, it has not been possible to establish their influence on participation by isolating the effect of other factors and hence more studies are needed to identify them.

## Supplementary data

Supplementary data are available at *EURPUB* online.

## Supplementary Material

ckaa203_Supplementary_Data
