# Supplementary material for: Inequalities and risk factors related to non-participation in colorectal cancer screening programmes: a systematic review
Source: Eur J Public Health. 2020 Dec 12;31(2):346–55. doi: 10.1093/eurpub/ckaa203 (PMC8071594; doi:10.1093/eurpub/ckaa203)
Supplement: ckaa203_Supplementary_Data [file ckaa203_supplementary_data.zip › ejph-2020-05-om-0502-File006.docx]

Supplementary Table 4. Results of quality assessment of the intervention study

| Study | Criteria | | | | | | | | | | | | | | Quality |
| --- | --- | --- | --- | --- | --- | --- | --- | --- | --- | --- | --- | --- | --- | --- | --- |
|  | 1 | 2 | 3 | 4 | 5 | 6 | 7 | 8 | 9 | 10 | 11 | 12 | 13 | 14 |  |
| Senore, 2010 | Y | Y | N | N | N | Y | NA | NA | Y | Y | Y | Y | Y | Y | Fair |

CD = Cannot determine, N = No, NA = Not applicable, NR = Not reported, Y = Yes.

1. Was the study described as randomized, a randomized trial, a randomized clinical trial, or an RCT?

2. Was the method of randomization adequate (i.e., use of randomly generated assignment)?

3. Was the treatment allocation concealed (so that assignments could not be predicted)?

4. Were study participants and providers blinded to treatment group assignment?

5. Were the people assessing the outcomes blinded to the participants' group assignments?

6. Were the groups similar at baseline on important characteristics that could affect outcomes (e.g., demographics, risk factors, co-morbid conditions)

7. Was the overall drop-out rate from the study at endpoint 20 or lower of the number allocated to treatment?

8. Was the differential drop-out rate (between treatment groups) at endpoint 15 percentage points or lower?

9. Was there high adherence to the intervention protocols for each treatment group?

10. Were other interventions avoided or similar in the groups (e.g., similar background treatments)?

11. Were outcomes assessed using valid and reliable measures, implemented consistently across all study participants?

12. Did the authors report that the sample size was sufficiently large to be able to detect a difference in the main outcome between groups with at least 80 power?

13. Were outcomes reported or subgroups analyzed prespecified (i.e., identified before analyses were conducted)?

14. Were all randomized participants analyzed in the group to which they were originally assigned, i.e., did they use an intention-to-treat analysis?
