# Supplementary material for: Inequalities and risk factors related to non-participation in colorectal cancer screening programmes: a systematic review
Source: Eur J Public Health. 2020 Dec 12;31(2):346–55. doi: 10.1093/eurpub/ckaa203 (PMC8071594; doi:10.1093/eurpub/ckaa203)
Supplement: ckaa203_Supplementary_Data [file ckaa203_supplementary_data.zip › ejph-2020-05-om-0502-File004.docx]

Supplementary Table 2. Excluded studies with reasons

| **Poor quality** |
| --- |
| Artama M, Heinavaara S, Sarkeala T, Prattala R, Pukkala E, Malila N. Determinants of non-participation in a mass screening program for colorectal cancer in Finland. Acta Oncol 2016;55:870-4.  Menvielle G, Dugas J, Richard J-, Luce D. Socioeconomic and healthcare use-related determinants of cervical, breast and colorectal cancer screening practice in the French West Indies. Eur J Cancer Prev 2018;27:269-73. |
| **High risk population** |
| Bostean G, Crespi CM, McCarthy WJ. Associations among family history of cancer, cancer screening and lifestyle behaviors: a population-based study. Cancer Causes & Control 2013;24:1491-503. |
| **45-75 year-old population** |
| Augustson EM, Vadaparampil ST, Paltoo DN, Kidd LR, O'Malley AS. Association between CBE, FOBT, and Pap smear adherence and mammography adherence among older low-income women. Prev Med 2003;36:734-9.  Gonzales M, Nelson H, Rhyne RL, Stone SN, Hoffman RM. Surveillance of colorectal cancer screening in New Mexico Hispanics and non-hispanic whites. J Community Health 2012;37:1279-88.  Gorin SS. Correlates of colorectal cancer screening compliance among urban Hispanics. J Behav Med 2005;28:125-37.  Gorin SS, Heck JE. Cancer screening among Latino subgroups in the United States. Prev Med 2005;40:515-26.  Greene P, Mehta P, Yeary KH, Bursac Z, Zhang J, Goldsmith G, et al. Using population data to reduce disparities in colorectal cancer screening, Arkansas, 2006. Prev Chronic Dis 2012;9:E138.  Griffith KA, McGuire DB, Royak-Schaler R, Plowden KO, Steinberger EK. Influence of family history and preventive health behaviors on colorectal cancer screening in African Americans. Cancer 2008;113:276-85.  Guerrero-Preston R, Chan C, Vlahov D, Mitchell MK, Johnson SB, Freeman H. Previous cancer screening behavior as predictor of endoscopic colon cancer screening among women aged 50 and over, in NYC 2002. J Community Health 2008;33:10-21. |
| He E, Lew JB, Egger S, Banks E, Ward RL, Beral V, et al. Factors associated with participation in colorectal cancer screening in Australia: Results from the 45 and Up Study cohort. Prev Med 2018;106:185-93.  Leone LA, Allicock M, Pignone MP, Johnson LS, Walsh JF, Campbell MK. Cancer screening patterns by weight group and gender for urban African American church members. J Community Health 2012;37:299-306.  Littman AJ, Koepsell TD, Forsberg CW, Haselkorn JK, Boyko EJ. Preventive services in veterans in relation to disability. J Rehabil Res Dev 2012;49:339-50.  Martinez KA, Pollack CE, Phelan DF, Markakis D, Bone L, Shapiro G, et al. Gender differences in correlates of colorectal cancer screening among black medicare beneficiaries in Baltimore. Cancer Epidemiol Biomarkers Prev 2013;22:1037-42.  Mauri D, Valachis A, Polyzos NP, Cortinovis I, Karampoiki V, Loukidou E, et al. Screening practice and misplaced priorities. Clin Transl Oncol 2009;11:228-36.  McQueen A, Vernon SW, Meissner HI, Klabunde CN, Rakowski W. Are there gender differences in colorectal cancer test use prevalence and correlates? Cancer Epidemiol Biomarkers Prev 2006;15:782-91.  Meissner HI, Breen N, Klabunde CN, Vernon SW. Patterns of colorectal cancer screening uptake among men and women in the United States. Cancer Epidemiol Biomarkers Prev 2006;15:389-94.  Meissner HI, Yabroff KR, Dodd KW, Leader AE, Ballard-Barbash R, Berrigan D. Are patterns of health behavior associated with cancer screening? Am J Health Promot 2009;23:168-75.  Owusu D, Quinn M, Wang KS. Alcohol consumption, depression, insomnia and colorectal cancer screening: Racial differences. Int J High Risk Behav Addict 2015;4:.  Peytremann-Bridevaux I, Voellinger R, Santos-Eggimann B. Healthcare and preventive services utilization of elderly Europeans with depressive symptoms. J Affective Disord 2008;105:247-52.  Seeff LC, Nadel MR, Klabunde CN, Thompson T, Shapiro JA, Vernon SW, et al. Patterns and predictors of colorectal cancer test use in the adult U.S. population. Cancer 2004;100:2093-103.  Shenson D, Adams M, Bolen J, Anderson L. Routine checkups don't ensure that seniors get preventive services. J Fam Pract 2011;60:E1-E10.  Simonds VW, Colditz GA, Rudd RE, Sequist TD. Cancer screening among Native Americans in California. Ethn Dis 2011;21:202-9. |
| Swan J, Breen N, Graubard BI, Mcneel TS, Blackman D, Tangka FK, et al. Data and trends in cancer screening in the United States: Results from the 2005 National Health Interview Survey. Cancer 2010;116:4872-81.  Tabbarah M, Nowalk MP, Raymund M, Jewell IK, Zimmerman RK. Barriers and facilitators of colon cancer screening among patients at faith-based neighborhood health centers. J Community Health 2005;30:55-74.  Tabuchi T, Murayama H, Hoshino T, Nakayama T. An Out-of-Pocket Cost Removal Intervention on Fecal Occult Blood Test Attendance. Am J Prev Med 2017;53:e51-62.  Weber MF, Cunich M, Smith DP, Salkeld G, Sitas F, O'Connell D. Sociodemographic and health-related predictors of self-reported mammogram, faecal occult blood test and prostate specific antigen test use in a large Australian study. BMC Public Health 2013;13:.  Wee LE, Cher WQ, Sin D, Li ZC, Koh GC-. Primary care characteristics and their association with health screening in a low-socioeconomic status public rental-flat population in Singapore-a mixed methods study Service organization, utilization, and delivery of care. BMC Fam Pract 2016;17:.  Wilcox ML, Acuña JM, de la Vega, P R., Castro G, Madhivanan P. Factors associated with compliance of blood stool test and use of colonoscopy in underserved communities of North Miami-Dade County, Florida. J Health Care Poor Underserved 2015;26:1319-35.  Withrow DR, Amartey A, Marrett LD. Cancer risk factors and screening in the off-reserve First Nations, Metis and non-Aboriginal populations of Ontario. Chronic Dis Inj Can 2014;34:1. |
| **Social inequities** |
| Aas E. Pecuniary compensation increases participation in screening for colorectal cancer. Health Econ 2009;18:337-54.  Al-Naggar RA, Al-Kubaisy W, Yap BW, Bobryshev YV, Osman MT. Attitudes towards colorectal cancer (CRC) and CRC screening tests among elderly Malay patients. Asian Pac J Cancer Preven 2015;16:667-74.  Brittain K, Murphy VP. Sociocultural and health correlates related to colorectal cancer screening adherence among urban African Americans. Cancer Nurs 2015;38:118-24.  Brouse CH, Basch CE, Wolf RL, Shmukler C, Neugut AI, Shea S. Barriers to colorectal cancer screening with fecal occult blood testing in a predominantly minority urban population: a qualitative study. Am J Public Health 2003;93:1268-71. |
| Brown SR, Joshweseoma L, Saboda K, Sanderson P, Ami D, Harris R. Cancer Screening on the Hopi Reservation: A Model for Success in a Native American Community. J Community Health 2015;**40:**1165-72.  Carlos RC, Fendrick AM, Patterson SK, Bernstein SJ. Associations in breast and colon cancer screening behavior in women. Acad Radiol 2005;**12:**451-8.  Christie J, Hooper C, Redd WH, Winkel G, Duhamel K, Itzkowitz S, et al. Predictors of endoscopy in minority women. J Natl Med Assoc 2005;**97:**1361-8.  Christy SM, Davis SN, Williams KR, Zhao X, Govindaraju SK, Quinn GP, et al. A community-based trial of educational interventions with fecal immunochemical tests for colorectal cancer screening uptake among blacks in community settings. Cancer 2016;**122:**3288-96.  Clark MA, Rogers ML, Armstrong GF, Rakowski W, Bowen DJ, Hughes T, et al. Comprehensive cancer screening among unmarried women aged 40-75 years: results from the cancer screening project for women. Journal of Women's Health 2009;**18:**451-9.  Crosby RA, Collins T. Correlates of Community-Based Colorectal Cancer Screening in a Rural Population: The Role of Fatalism. Journal of Rural Health 2017;**33:**402-5.  Czaderny K. Increasing deaths from colorectal cancer in Poland - Insights for optimising colorectal cancer screening in society and space. Annals of Agricultural and Environmental Medicine 2019;**26:**125-32.  Eichholzer M, Richard A, Rohrmann S, Schmid SM, Leo C, Huang DJ, et al. Breast cancer screening attendance in two Swiss regions dominated by opportunistic or organized screening. BMC Health Serv Res 2016;**16:**.  Fedewa SA, Goodman M, Flanders WD, Han X, Smith RA, Ward M, et al. Elimination of cost-sharing and receipt of screening for colorectal and breast cancer. Cancer 2015;**121:**3272-80.  Gonzalez P, Castaneda SF, Mills PJ, Talavera GA, Elder JP, Gallo LC. Determinants of breast, cervical and colorectal cancer screening adherence in Mexican-American women. J Community Health 2012;**37:**421-33.  Green BB, Bogart A, Chubak J, Vernon SW, Morales LS, Meenan RT, et al. Nonparticipation in a population-based trial to increase colorectal cancer screening. Am J Prev Med 2012;**42:**390-7.  Greiner KA, Daley CM, Epp A, James A, Yeh HW, Geana M, et al. Implementation intentions and colorectal screening: A randomized trial in safety-net clinics. Am J Prev Med 2014;**47:**703-14. |
| Guiriguet C, Pera G, Castells A, Toran P, Grau J, Rivero I, et al. Impact of comorbid conditions on participation in an organised colorectal cancer screening programme: A cross-sectional study. BMC Cancer 2017;**17:**524.  Harmon BE, Little MA, Woekel ED, Ettienne R, Long CR, Wilkens LR, et al. Ethnic differences and predictors of colonoscopy, prostate-specific antigen, and mammography screening participation in the multiethnic cohort. Cancer Epidemiology 2014;**38:**162-7.  Hoffmeister M, Holleczek B, Stock C, Zwink N, Stolz T, Stegmaier C, et al. Utilization and determinants of follow-up colonoscopies within 6 years after screening colonoscopy: Prospective cohort study. International Journal of Cancer 2019;**144:**402-10.  Long MD, Lance T, Robertson D, Kahwati L, Kinsinger L, Fisher DA. Colorectal cancer testing in the national veterans health administration. Dig Dis Sci 2012;**57:**288-93.  Longacre AV, Cramer LD, Gross CP. Screening colonoscopy use among individuals at higher colorectal cancer risk. J Clin Gastroenterol 2006;**40:**490-6.  Lopez-Charneco M, Perez CM, Soto-Salgado M, Rodriguez L, Gonzalez D, Serrano R, et al. Correlates of colorectal cancer screening among Hispanics: Results from the 2008 Puerto Rico behavioral risk factor surveillance system survey. P R Health Sci J 2013;**32:**68-75.  Mankaney G, Rizk M, Sarvepalli S, Bongorno J, Garber A, Lopez R, et al. Patient-Initiated Colonoscopy Scheduling Effectively Increases Colorectal Cancer Screening Adherence. Dig Dis Sci 2019;agnaton.  Manne S, Steinberg MB, Delnevo C, Ulpe R, Sorice K. Colorectal Cancer Screening Among Foreign-born South Asians in the Metropolitan New York/New Jersey Region. J Community Health 2015;**40:**1075-83.  Mitchell RS, Padwal RS, Chuck AW, Klarenbach SW. Cancer screening among the overweight and obese in Canada. Am J Prev Med 2008;**35:**127-32.  Muus KJ, Baker-Demaray T, McDonald LR, Ludtke RL, Allery AJ, Bogart TA, et al. Body mass index and cancer screening in older American Indian and Alaska Native men. Journal of Rural Health 2009;**25:**104-8.  Myong JP, Shin JY, Kim SJ. Factors associated with participation in colorectal cancer screening in Korea: The Fourth Korean National Health and Nutrition Examination Survey (KNHANES IV). Int J Colorectal Dis 2012;**27:**1061-9.  O'Malley AS, Forrest CB, Mandelblatt J. Adherence of low-income women to cancer screening recommendations. Journal of General Internal Medicine 2002;**17:**144-54. |
| Park SM, Park CT, Park SY, Bae DS, Nam JH, Cho CH, et al. Factors related to second cancer screening practice in disease-free cervical cancer survivors. Cancer Causes and Control 2009;**20:**1697-703.  Phillips KL, Smith ML, Ahn S, Ory MG, Hochhalter AK. Correlates of initiating colorectal cancer screening beginning at age 50. J Community Health 2013;**38:**23-30.  Ricardo-Rodrigues I, Jimenez-Garcia R, Hernandez-Barrera V, Carrasco-Garrido P, Jimenez-Trujillo I, Lopez-de-Andres A. Adherence to and predictors of participation in colorectal cancer screening with faecal occult blood testing in Spain, 2009-2011. European Journal of Cancer Prevention 2015;**24:**305-12.  Sentell TL, Tsoh JY, Davis T, Davis J, Braun KL. Low health literacy and cancer screening among Chinese Americans in California: a cross-sectional analysis. BMJ Open 2015;**5:**e006104.  Shippee ND, Mullan RJ, Nabhan M, Kermott CA, Hagen PT, Rhodes DJ, et al. Adherence to preventive recommendations: experience of a cohort presenting for executive health care. Population Health Management 2012;**15:**65-70.  Stanley SL, King JB, Thomas CC, Richardson LC. Factors associated with never being screened for colorectal cancer. J Community Health 2013;**38:**31-9.  Stockwell DH, Woo P, Jacobson BC, Remily R, Syngal S, Wolf J, et al. Determinants of colorectal cancer screening in women undergoing mammography. Am J Gastroenterol 2003;**98:**1875-80.  Thorpe LE, Mostashari F, Hajat A, Nash D, Karpati A, Weber T, et al. Colon cancer screening practices in New York city, 2003: Results of a large random-digit dialed telephone survey. Cancer 2005;**104:**1075-82.  Venturelli F, Sampaolo L, Carrozzi G, Zappa M, Giorgi Rossi P, Group PW. Associations between cervical, breast and colorectal cancer screening uptake, chronic diseases and health-related behaviours: Data from the Italian PASSI nationwide surveillance. Prev Med 2019;**120:**60-70.  Williams L, Looney S, Joshua T, McCall A, Tingen M. Demographic, Psychosocial, and Behavioral Associations with Cancer Screening Among a Homeless Population. Journal of Thoracic Oncology 2018;**Conference:**Otober.  Young WF, McGloin J, Zittleman L, West DR, Westfall JM. Predictors of colorectal screening in rural Colorado: testing to prevent colon cancer in the high plains research network. Journal of Rural Health 2007;**23:**238-45. |
| **Preventive activities or CRC risk-factors** |
| Adonis L, Basu D, Luiz J. Predictors of adherence to screening guidelines for chronic diseases of lifestyle, cancers, and HIV in a health-insured population in South Africa. Global Health Action 2014;**7:**1-8.  Ahluwalia IB, Bolen J, Garvin B. Health insurance coverage and use of selected preventive services by working-age women, BRFSS, 2006. Journal of Women's Health 2007;**16:**935-40.  Almadi MA, Mosli MH, Bohlega MS, Al Essa MA, AlDohan MS, Alabdallatif TA, et al. Effect of public knowledge, attitudes, and behavior on willingness to undergo colorectal cancer screening using the health belief model. Saudi Journal of Gastroenterology 2015;**21:**71-7.  Balluz L, Ahluwalia IB, Murphy W, Mokdad A, Giles W, Harris VB. Surveillance for certain health behaviors among selected local areas--United States, Behavioral Risk Factor Surveillance System, 2002. MMWR Surveill Summ 2004;**53:**1-100.  Bardach SH, Schoenberg NE, Fleming ST, Hatcher J. Relationship Between Colorectal Cancer Screening Adherence and Knowledge Among Vulnerable Rural Residents of Appalachian Kentucky. Cancer Nurs 2012;**35:**288-94.  Bazargan M, Ani C, Bazargan-Hejazi S, Baker RS, Bastani R. Colorectal cancer screening among underserved minority population: discrepancy between physicians' recommended, scheduled, and completed tests. Patient Education {\&} Counseling 2009;**76:**240-7.  Bolen JC, Rhodes L, Powell-Griner EE, Bland SD, Holtzman D. State-specific prevalence of selected health behaviors, by race and ethnicity--Behavioral Risk Factor Surveillance System, 1997. MMWR.CDC surveillance summaries : Morbidity and mortality weekly report.CDC surveillance summaries / Centers for Disease Control 2000;**49:**1-60.  Braun KL, Fong M, Kaanoi ME, Kamaka ML, Gotay CC. Testing a culturally appropriate, theory-based intervention to improve colorectal cancer screening among Native Hawaiians. Prev Med 2005;**40:**619-27.  Brouse CH, Basch CE, Wolf RL, Shmukler C. Barriers to colorectal cancer screening in a low income, urban population: A descriptive study. Health Educ 2004;**104:**68-76.  Carrasco-Garrido P, Hernandez-Barrera V, Lopez de AA, Jimenez-Trujillo I, Gallardo PC, Jimenez-Garcia R. Awareness and uptake of colorectal, breast, cervical and prostate cancer screening tests in Spain. Eur J Public Health 2014;**24:**264-70. |
| Carrozzi G, Sampaolo L, Bolognesi L, Sardonini L, Bertozzi N, Rossi PG, et al. Cancer screening uptake: Association with individual characteristics, geographic distribution, and time trends in Italy. Epidemiol Prev 2015;**39:**9-18.  Choi KC, So WK, Chan DN, Shiu AT, Ho SS, Chan HY, et al. Gender differences in the use of colorectal cancer tests among older Chinese adults. European Journal of Oncology Nursing 2013;**17:**603-9.  Choi KC, So WK, Chen JM, Lau GC, Lee PC, Chan CW. Comparison Study of Uptake of Colorectal Cancer Testing between Ethnic Minorities and the General Population in Hong Kong. Asian Pacific Journal of Cancer Prevention: Apjcp 2015;**16:**7713-20.  Cullati S, Charvet-Bérard AI, Perneger TV. Cancer screening in a middle-aged general population: Factors associated with practices and attitudes. BMC Public Health 2009;**9:**.  Dassow P. Setting educational priorities for women's preventive health: Measuring beliefs about screening across disease states. Journal of Women's Health 2005;**14:**324-30.  Deshpande AD, McQueen A, Coups EJ. Different effects of multiple health status indicators on breast and colorectal cancer screening in a nationally representative US sample. Cancer Epidemiology 2012;**36:**270-5.  Domingo JB, Chen JJ, Braun KL. Colorectal Cancer Screening Compliance among Asian and Pacific Islander Americans. Journal of Immigrant {\&} Minority Health 2018;**20:**584-93.  Dubard CA, Schmid D, Yow A, Rogers AB, Lawrence WW. Recommendation for and receipt of cancer screenings among medicaid recipients 50 years and older. Arch Intern Med 2008;**168:**2014-21.  Dulai GS, Farmer MM, Ganz PA, Bernaards CA, Qi K, Dietrich AJ, et al. Primary Care Provider Perceptions of Barriers to and Facilitators of Colorectal Cancer Screening in a Managed Care Setting. Cancer 2004;**100:**1.  Fagerlin A, Sepucha KR, Couper MP, Levin CA, Singer E, Zikmund-Fisher BJ. Patients' knowledge about 9 common health conditions: The DECISIONS survey. Med Decis Mak 2010;**30:**35S-52S.  Farraye FA, Wong M, Hurwitz S, Puleo E, Emmons K, Wallace MB, et al. Barriers to endoscopic colorectal cancer screening: are women different from men? Am J Gastroenterol 2004;**99:**341-9.  Felsen CB, Piasecki A, Ferrante JM, Ohman-Strickland PA, Crabtree BF. Colorectal cancer screening among primary care patients: does risk affect screening behavior? J Community Health 2011;**36:**605-11. |
| Fernandez ME, Wippold R, Torres-Vigil I, Byrd T, Freeberg D, Bains Y, et al. Colorectal cancer screening among Latinos from U.S. cities along the Texas-Mexico border. Cancer Causes {\&} Control 2008;**19:**195-206.  Ferrat E, Le BJ, Veerabudun K, Bercier S, Brixi Z, Khoshnood B, et al. Colorectal cancer screening: factors associated with colonoscopy after a positive faecal occult blood test. Br J Cancer 2013;**109:**1437-44.  Friedman LC, Puryear LJ, Moore A, Green CE. Breast and colorectal cancer screening among low-income women with psychiatric disorders. Psychooncology 2005;**14:**786-91.  Friedman LC, Webb JA, Everett TE. Psychosocial and medical predictors of colorectal cancer screening among low-income medical outpatients. Journal of Cancer Education 2004;**19:**180-6.  Fujiwara M, Inagaki M, Nakaya N, Fujimori M, Higuchi Y, Hayashibara C, et al. Cancer screening participation in schizophrenic outpatients and the influence of their functional disability on the screening rate: A cross-sectional study in Japan. Psychiatry Clin Neurosci 2017;**71:**813-25.  Fujiwara M, Inagaki M, Nakaya N, Fujimori M, Higuchi Y, Kakeda K, et al. Association between serious psychological distress and nonparticipation in cancer screening and the modifying effect of socioeconomic status: Analysis of anonymized data from a national cross-sectional survey in Japan. Cancer 2018;**124:**555-62.  Fukuda Y, Nakamura K, Takano T. Reduced likelihood of cancer screening among women in urban areas and with low socio-economic status: A multilevel analysis in Japan. Public Health 2005;**119:**875-84.  Gobl CS, Ortag F, Bozkurt L, Smeikal A, Dadak C, Kautzky-Willer A. Health behaviour and attitude towards screening examinations in an Austrian urban and rural population: gender aspects - screening and sex. Wiener Medizinische Wochenschrift 2011;**161:**143-8.  Goel MS, Wee CC, McCarthy EP, Davis RB, Ngo-Metzger Q, Phillips RS. Racial and Ethnic Disparities in Cancer Screening: The Importance of Foreign Birth as a Barrier to Care. Journal of General Internal Medicine 2003;**18:**1028-35.  Hatano Y, Matsumoto M, Inoue K, Takeuchi K. Rurality and participation in mass preventive health services: A nationwide descriptive study. Hiroshima J Med Sci 2013;**62:**43-8.  Hatcher J, Dignan MB, Schoenberg N. How do rural health care providers and patients view barriers to colorectal cancer screening? Insights from appalachian kentucky. Nurs Clin North Am 2011;**46:**181-92. |
| Hozawa A, Kuriyama S, Watanabe I, Kakizaki M, Ohmori-Matsuda K, Sone T, et al. Participation in health check-ups and mortality using propensity score matched cohort analyses. Prev Med 2010;**51:**397-402.  Inagaki M, Fujiwara M, Nakaya N, Fujimori M, Higuchi Y, Hayashibara C, et al. Low Cancer Screening Rates among Japanese People with Schizophrenia: A Cross-Sectional Study. Tohoku J Exp Med 2018;**244:**209-18.  Jandorf L, Ellison J, Villagra C, Winkel G, Varela A, Quintero-Canetti Z, et al. Understanding the barriers and facilitators of colorectal cancer screening among low income immigrant hispanics. Journal of Immigrant {\&} Minority Health 2010;**12:**462-9.  Kim S, Song JH, Oh YM, Park SM. Disparities in the utilisation of preventive health services by the employment status: An analysis of 2007-2012 South Korean national survey. PLoS ONE 2018;**13:**e0207737.  Kwon JS, Elit L, Saskin R, Hodgson D, Grunfeld E. Secondary cancer prevention during follow-up for endometrial cancer. Obstet Gynecol 2009;**113:**790-5.  Lee HY, Park EC, Jun JK, Hahm MI, Jung KW, Kim Y, et al. Trends in Socioeconomic Disparities in Organized and Opportunistic Gastric Cancer Screening in Korea (2005-2009). Cancer Epidemiology Biomarkers {\&} Prevention 2010;**19:**1919-26.  Lee SM, Chen L, Jung MY, Baezconde-Garbanati L, Juon HS. Acculturation and Cancer Screening Among Asian Americans: Role of Health Insurance and Having a Regular Physician. J Community Health 2014;**39:**201-12.  Lees KA, Wortley PM, Coughlin SS. Comparison of Racial/Ethnic Disparities in Adult Immunization and Cancer Screening. Am J Prev Med 2005;**29:**404-11.  Maly AG, Steel TL, Fu R, Lieberman DA, Becker TM. Colorectal cancer screening among American Indians in a Pacific Northwest tribe: Cowlitz tribal BRFSS project, 2009-2010. Public Health Rep 2014;**129:**280-8.  Mann BD, Sherman L, Clayton C, Johnson RF, Keates J, Kasenge R, et al. Screening to the converted: An educational intervention in African American churches. Journal of Cancer Education 2000;**15:**46-50.  Massetti GM, Townsend JS, Thomas CC, Basile KC, Richardson LC. Healthcare Access and Cancer Screening among Victims of Intimate Partner Violence. Journal of Women's Health 2018;**27:**607-14. |
| Maxwell AE, Bastani R, Warda US. Demographic predictors of cancer screening among Filipino and Korean immigrants in the United States. Am J Prev Med 2000;**18:**62-8.  May FP, Whitman CB, Varlyguina K, Bromley EG, Spiegel BMR. Addressing Low Colorectal Cancer Screening in African Americans: Using Focus Groups to Inform the Development of Effective Interventions. Journal of Cancer Education 2016;**31:**567-74.  Mayer DK, Terrin NC, Menon U, Kreps GL, McCance K, Parsons SK, et al. Screening practices in cancer survivors. Journal of Cancer Survivorship 2007;**1:**17-26.  Mojica CM, Flores B, Ketchum NS, Liang Y. Health Care Access, Utilization, and Cancer Screening Among Low-Income Latina Women. Hispanic Health Care International : The Official Journal of The National Association of Hispanic Nurses 2017;**15:**160-5.  Narayan AK, Flores EJ, Harvey HB, Lehman CD. Population-Based Health Engagement Opportunities Through Breast Imaging: A Population-Based Cross-Sectional Survey. J Am Coll Radiol 2018;**15:**1401-7.  Nathan PC, Ness KK, Mahoney MC, Li Z, Hudson MM, Ford JS, et al. Screening and surveillance for second malignant neoplasms in adult survivors of childhood cancer: A report from the childhood cancer survivor study. Ann Intern Med 2010;**153:**442-51.  Nicholls R, Perry L, Gallagher R, Duffield C, Sibbritt D, Xu XY. The personal cancer screening behaviours of nurses and midwives. J Adv Nurs 2017;**73:**1403-20.  Niv Y, Lev-El M, Fraser G, Abuksis G, Tamir A. Protective effect of faecal occult blood test screening for colorectal cancer: Worse prognosis for screening refusers. Gut 2002;**50:**2002.  O'Donnell S, Goldstein B, DiMatteo MR, Fox SA, John CR, Obrzut JE. Adherence to mammography and colorectal cancer screening in women 50-80 years of age: The role of psychological distress. Womens Health Issues 2010;**20:**343-9.  O'Malley AS, Beaton E, Yabroff KR, Abramson R, Mandelblatt J. Patient and provider barriers to colorectal cancer screening in the primary care safety-net. Prev Med 2004;**39:**56-63.  Perkins A, Nicholls K, Shaw T, Liu G, Molokhia E. Attitudes toward colorectal cancer screening in the digital age: a survey of practices and attitudes among screening-eligible Alabamians. South Med J 2013;**106:**462-7.  Potter MB, Gildengorin G, Wang Y, Wu M, Kroon L. Comparative effectiveness of two pharmacy-based colorectal cancer screening interventions during an annual influenza vaccination campaign. Journal of the American Pharmacists Association 2010;**50:**181-7. |
| Rogers CR, Robinson CD, Arroyo C, Obidike OJ, Sewali B, Okuyemi KS. Colorectal Cancer Screening Uptake's Association With Psychosocial and Sociodemographic Factors Among Homeless Blacks and Whites. Health Education {\&} Behavior 2017;**44:**928-36.  Roussel C, Touboul C. Large population survey: Strengths and limits. Methodology of the EDIFICE survey. European Journal of Cancer Prevention 2011;**20:**S5-7.  Schapira MM, Neuner J, Fletcher KE, Gilligan MA, Hayes E, Laud P. The relationship of health numeracy to cancer screening. Journal of Cancer Education 2011;**26:**103-10.  Shahidi NC, Homayoon B, Cheung WY. Factors associated with suboptimal colorectal cancer screening in US immigrants. American Journal of Clinical Oncology 2013;**36:**381-7.  Shen SX, Lofters A, Tinmouth J, Paszat L, Rabeneck L, Glazier RH. Predictors of non-adherence to colorectal cancer screening among immigrants to Ontario, Canada: a population-based study. Prev Med 2018;**111:**180-9.  Van Tze CN, Fitzgerald H, Qureshi A, Tan HJ, Low ML. Pioneering annual colorectal cancer screening and treatment targeting low income communities in Malaysia (2010-2015). Asian Pacific Journal of Cancer Prevention 2016;**17:**3179-83.  Waters EA, Kiviniemi MT, Orom H, Hay JL. I don't know my cancer risk: Implications for health behavior engagement. Annals of Behavioral Medicine 2016;**50:**784-8.  Wee CC, McCarthy EP, Phillips RS. Factors associated with colon cancer screening: The role of patient factors and physician counseling. Prev Med 2005;**41:**23-9.  Wilf-Miron R, Peled R, Yaari E, Vainer A, Porath A, Kokia E. The association between socio-demographic characteristics and adherence to breast and colorectal cancer screening: analysis of large sub populations. BMC Cancer 2011;**11:**376.  Willems B, Bracke P. The education gradient in cancer screening participation: a consistent phenomenon across Europe? Int J Public Health 2018;**63:**93-103. |
