# Supplementary material for: Inequalities and risk factors related to non-participation in colorectal cancer screening programmes: a systematic review
Source: Eur J Public Health. 2020 Dec 12;31(2):346–55. doi: 10.1093/eurpub/ckaa203 (PMC8071594; doi:10.1093/eurpub/ckaa203)
Supplement: ckaa203_Supplementary_Data [file ckaa203_supplementary_data.zip › ejph-2020-05-om-0502-File003.docx]

Supplementary Table 1. Medline search strategy

| **#** | **Search** | **Hits** |
| --- | --- | --- |
| 1 | Diabetes Mellitus/ | 111,773 |
| 2 | Metabolic Syndrome/ | 29,059 |
| 3 | Obesity/ | 168,462 |
| 4 | Periodontal Diseases/ | 25,464 |
| 5 | Gingivitis/ | 9,551 |
| 6 | Periodontitis/ | 16,960 |
| 7 | 4 or 5 or 6 | 47,416 |
| 8 | Hyperlipidemias/ | 26,353 |
| 9 | Hypertension/ | 225,551 |
| 10 | Inflammatory Bowel Diseases/ | 19,864 |
| 11 | Influenza Vaccines/ | 21,502 |
| 12 | Exercise/ | 98,747 |
| 13 | Sedentary Behavior/ | 7,732 |
| 14 | Alcohol Drinking/ | 63,578 |
| 15 | "Tobacco Use"/ | 1,204 |
| 16 | Tobacco Smoking/ | 488 |
| 17 | Red Meat/ | 1,954 |
| 18 | Dietary Carbohydrates/ | 25,104 |
| 19 | highly refined carbohydrates.mp. | 5 |
| 20 | highly refined grains.mp. | 2 |
| 21 | health care utili$ation.mp. | 0 |
| 22 | "Patient Acceptance of Health Care"/ | 42,040 |
| 23 | health care utilisation.mp. | 446 |
| 24 | healthcare utilization.mp. | 2,612 |
| 25 | healthcare utilisation.mp. | 528 |
| 26 | Health Services Misuse/ | 4,225 |
| 27 | Attitude to Health/ | 81,478 |
| 28 | Breast Neoplasms/ | 269,415 |
| 29 | breast tumour*.mp. | 3,199 |
| 30 | breast adenocarcinoma*.mp. | 2,217 |
| 31 | breast adeno-carcinoma*.mp. | 8 |
| 32 | breast adenoma*.mp. | 32 |
| 33 | Uterine Cervical Neoplasms/ | 71,420 |
| 34 | cervical tumo?r*.mp. | 1,466 |
| 35 | cervical tumor*.mp. | 1,259 |
| 36 | cervical tumour*.mp. | 208 |
| 37 | 35 or 36 | 1,466 |
| 38 | cervical carcinoma*.mp. | 10,354 |
| 39 | cervix carcinoma*.mp. | 1,318 |
| 40 | cervical adenocarcinoma*.mp. | 1,159 |
| 41 | cervix adenocarcinoma*.mp. | 126 |
| 42 | cervical adeno-carcinoma*.mp. | 1 |
| 43 | cervix adeno-carcinoma*.mp. | 0 |
| 44 | cervical adenoma*.mp. | 15 |
| 45 | cervix adenoma*.mp. | 4 |
| 46 | 1 or 2 or 3 or 4 or 5 or 6 or 8 or 9 or 10 or 11 or 12 or 13 or 14 or 15 or 16 or 17 or 18 or 19 or 20 or 22 or 23 or 24 or 25 or 26 or 27 or 28 or 29 or 30 or 31 or 32 or 33 or 34 or 38 or 39 or 40 or 41 or 42 or 43 or 44 or 45 | 1,219,094 |
| 47 | Colorectal Neoplasms/ | 79,768 |
| 48 | Rectal Neoplasms/ | 40,231 |
| 49 | Colonic Neoplasms/ | 68,221 |
| 50 | (colorectal or colon or colonic or bowel or rectal or rectum or sigmoid or anal or anus).mp. [mp=title, abstract, original title, name of substance word, subject heading word, floating sub-heading word, keyword heading word, organism supplementary concept word, protocol supplementary concept word, rare disease supplementary concept word, unique identifier, synonyms] | 519,842 |
| 51 | (cancer* or neoplasm* or tumor* or tumour* or carcinoma* or adenocarcinoma* or adeno-carcinoma* or adenoma* or lesion or malignan*).mp. [mp=title, abstract, original title, name of substance word, subject heading word, floating sub-heading word, keyword heading word, organism supplementary concept word, protocol supplementary concept word, rare disease supplementary concept word, unique identifier, synonyms] | 3,675,893 |
| 52 | 50 and 51 | 282,525 |
| 53 | 47 or 48 or 49 or 52 | 282,525 |
| 54 | Mass Screening/ | 97,165 |
| 55 | Population Surveillance/ | 56,454 |
| 56 | (direct-to-consumer screening and testing).mp. [mp=title, abstract, original title, name of substance word, subject heading word, floating sub-heading word, keyword heading word, organism supplementary concept word, protocol supplementary concept word, rare disease supplementary concept word, unique identifier, synonyms] | 110 |
| 57 | (direct to consumer screening and testing).mp. [mp=title, abstract, original title, name of substance word, subject heading word, floating sub-heading word, keyword heading word, organism supplementary concept word, protocol supplementary concept word, rare disease supplementary concept word, unique identifier, synonyms] | 110 |
| 58 | Early Diagnosis/ | 24,203 |
| 59 | early detection.mp. | 65,763 |
| 60 | Secondary Prevention/ | 18,921 |
| 61 | organized screening.mp. | 471 |
| 62 | organised screening.mp. | 220 |
| 63 | opportunistic screening.mp. | 831 |
| 64 | population based screening.mp. | 1,376 |
| 65 | population-based screening.mp. | 1,376 |
| 66 | 54 or 55 or 56 or 57 or 58 or 59 or 60 or 61 or 62 or 63 or 64 or 65 | 246,101 |
| 67 | participation.mp. or Patient Participation/ or Community Participation/ or Stakeholder Participation/ | 150,822 |
| 68 | Refusal to Participate/ | 590 |
| 69 | Voluntary Programmes/ | 1,416 |
| 70 | Patient Compliance/ | 55,479 |
| 71 | adherence.mp. | 122,027 |
| 72 | barrier*.mp. | 221,235 |
| 73 | facilitator*.mp. | 18,641 |
| 74 | research intervention*.mp. | 369 |
| 75 | invitation coverage.mp. | 5 |
| 76 | uptake.mp. | 325,868 |
| 77 | 67 or 68 or 69 or 70 or 71 or 72 or 73 or 74 or 75 or 76 | 839,068 |
| 78 | Socioeconomic Factors/ or Social Class/ | 175,981 |
| 79 | socioeconomics.mp. | 416 |
| 80 | socioeconomic level.mp. | 1,352 |
| 81 | socioeconomic position*.mp. | 2,333 |
| 82 | socioeconomic variation*.mp. | 186 |
| 83 | socioeconomic difference*.mp. | 1,254 |
| 84 | social status.mp. | 4,830 |
| 85 | social gradient.mp. | 541 |
| 86 | socioeconomic inequalit*.mp. [mp=title, abstract, original title, name of substance word, subject heading word, floating sub-heading word, keyword heading word, organism supplementary concept word, protocol supplementary concept word, rare disease supplementary concept word, unique identifier, synonyms] | 1,582 |
| 87 | socioeconomic inequit*.mp. [mp=title, abstract, original title, name of substance word, subject heading word, floating sub-heading word, keyword heading word, organism supplementary concept word, protocol supplementary concept word, rare disease supplementary concept word, unique identifier, synonyms] | 117 |
| 88 | socioeconomic equalit*.mp. [mp=title, abstract, original title, name of substance word, subject heading word, floating sub-heading word, keyword heading word, organism supplementary concept word, protocol supplementary concept word, rare disease supplementary concept word, unique identifier, synonyms] | 5 |
| 89 | socioeconomic equit*.mp. [mp=title, abstract, original title, name of substance word, subject heading word, floating sub-heading word, keyword heading word, organism supplementary concept word, protocol supplementary concept word, rare disease supplementary concept word, unique identifier, synonyms] | 21 |
| 90 | health disparit*.mp. [mp=title, abstract, original title, name of substance word, subject heading word, floating sub-heading word, keyword heading word, organism supplementary concept word, protocol supplementary concept word, rare disease supplementary concept word, unique identifier, synonyms] | 8,185 |
| 91 | Health Status Disparities/ | 13,481 |
| 92 | health care disparit*.mp. [mp=title, abstract, original title, name of substance word, subject heading word, floating sub-heading word, keyword heading word, organism supplementary concept word, protocol supplementary concept word, rare disease supplementary concept word, unique identifier, synonyms] | 901 |
| 93 | Healthcare Disparities/ | 14,283 |
| 94 | health inequalit*.mp. [mp=title, abstract, original title, name of substance word, subject heading word, floating sub-heading word, keyword heading word, organism supplementary concept word, protocol supplementary concept word, rare disease supplementary concept word, unique identifier, synonyms] | 4,145 |
| 95 | health inequit*.mp. [mp=title, abstract, original title, name of substance word, subject heading word, floating sub-heading word, keyword heading word, organism supplementary concept word, protocol supplementary concept word, rare disease supplementary concept word, unique identifier, synonyms] | 1,273 |
| 96 | health equalit*.mp. [mp=title, abstract, original title, name of substance word, subject heading word, floating sub-heading word, keyword heading word, organism supplementary concept word, protocol supplementary concept word, rare disease supplementary concept word, unique identifier, synonyms] | 74 |
| 97 | Health Equity/ | 740 |
| 98 | Education/ | 20,198 |
| 99 | educational level.mp. or Educational Status/ | 58,262 |
| 100 | Income/ | 27,364 |
| 101 | Poverty/ | 35,005 |
| 102 | deprivation.mp. | 74,403 |
| 103 | Employment/ | 44,051 |
| 104 | Occupations/ | 22,310 |
| 105 | Ethnic Groups/ | 58,272 |
| 106 | gender.mp. | 258,250 |
| 107 | gender inequalit*.mp. [mp=title, abstract, original title, name of substance word, subject heading word, floating sub-heading word, keyword heading word, organism supplementary concept word, protocol supplementary concept word, rare disease supplementary concept word, unique identifier, synonyms] | 967 |
| 108 | gender difference*.mp. | 25,827 |
| 109 | 106 or 107 or 108 | 258,250 |
| 110 | Rural Population/ or rural area*.mp. | 74,559 |
| 111 | Urban Population/ or urban area*.mp. | 73,208 |
| 112 | 78 or 79 or 80 or 81 or 82 or 83 or 84 or 85 or 86 or 87 or 88 or 89 or 90 or 91 or 92 or 93 or 94 or 95 or 96 or 97 or 98 or 99 or 100 or 101 or 102 or 103 or 104 or 105 or 106 or 107 or 108 or 110 or 111 | 785,024 |
| 113 | 46 and 53 and 66 and 77 and 112 | 259 |
| 114 | limit 113 to (yr="2000 -Current" and (english or french or portuguese or spanish)) | 243 |
